# Brain Age as a Biomarker in Alzheimer’s Disease: Narrative Perspectives on Imaging, Biomarkers, Machine Learning, and Intervention Potential

**DOI:** 10.3390/brainsci16010033

**Published:** 2025-12-25

**Authors:** Lan Lin, Yanxue Li, Shen Sun, Jeffery Lin, Ziyi Wang, Yutong Wu, Zhenrong Fu, Hongjian Gao

**Affiliations:** 1 Department of Biomedical Engineering, College of Chemistry and Life Science, Beijing University of Technology, Beijing 100124, China; lanlin@bjut.edu.cn (L.L.); liyanxue@emails.bjut.edu.cn (Y.L.); sunshen@bjut.edu.cn (S.S.); 2 Intelligent Physiological Measurement and Clinical Translation, Beijing International Base for Scientific and Technological Cooperation, Beijing University of Technology, Beijing 100124, China; 3 Paul G. Allen School of Computer Science & Engineering, University of Washington, Seattle, WA 98195, USA; jeffery1@uw.edu; 4 Information School, University of Washington, Seattle, WA 98195, USA; ziyiw88@uw.edu; 5 Key Laboratory of Adolescent Cyber Psychology and Behavior (CCNU), Ministry of Education, Wuhan 430079, China; yutong.wu@mails.ccnu.edu.cn; 6 Key Laboratory of Human Development and Mental Health of Hubei Province, School of Psychology, Central China Normal University, Wuhan 430079, China

**Keywords:** Alzheimer’s disease, brain age, Brain Age Gap, machine learning, amyloid/tau/neurodegeneration framework, longitudinal study, multimodal imaging

## Abstract

**Background/Objectives**: Alzheimer’s disease (AD) has a prolonged preclinical phase and marked heterogeneity. Brain age and the Brain Age Gap (BAG), derived from neuroimaging and machine learning (ML), offer a non-invasive, system-level indicator of brain integrity, with potential relevance for early detection, risk stratification, and intervention monitoring. This review summarizes the conceptual basis, imaging characteristics, biological relevance, and explores its potential clinical utility of BAG across the AD continuum. **Methods**: We conducted a narrative synthesis of evidence from morphometric structural magnetic resonance imaging (sMRI), connectivity-based functional magnetic resonance imaging (fMRI), positron emission tomography (PET), and diffusion tensor imaging (DTI), alongside recent advances in deep learning architectures and multimodal fusion techniques. We further examined associations between BAG and the Amyloid/Tau/Neurodegeneration (A/T/N) framework, neuroinflammation, cognitive reserve, and lifestyle interventions. **Results**: BAG may reflect neurodegeneration associated with AD, showing greater deviations in individuals with mild cognitive impairment (MCI) and early AD, and is correlated with tau pathology, neuroinflammation, and metabolic or functional network dysregulation. Multimodal and deep learning approaches enhance the sensitivity of BAG to disease-related deviations. Longitudinal BAG changes outperform static BAG in forecasting cognitive decline, and lifestyle or exercise interventions can attenuate BAG acceleration. **Conclusions**: BAG emerges as a promising, dynamic, integrative, and modifiable complementary biomarker with the potential for assessing neurobiological resilience, disease staging, and personalized intervention monitoring in AD. While further standardization and large-scale validation are essential to support clinical translation, BAG provides a novel systems-level perspective on brain health across the AD continuum.

## 1. Introduction

Alzheimer’s disease (AD) is a major public health challenge, characterized by a protracted preclinical phase followed by progressive cognitive and functional decline [1]. The current diagnostic paradigm—relying on late-stage symptoms and costly molecular biomarkers such as cerebrospinal fluid assays or amyloid (A)/tau (T) PET—offers limited feasibility for broad early detection [2,3]. These constraints highlight the need for scalable, non-invasive biomarkers. Such biomarkers should capture subtle preclinical neurodegenerative changes and differentiate healthy from pathological aging. The emergence of brain age, a metric derived from neuroimaging data using machine learning (ML) models, offers a systems-level approach to quantifying individualized brain health, and may help address the need for comprehensive neurobiological staging [4].

The conceptual foundation of the brain age framework lies in the quantification of the Brain Age Gap (BAG), defined as the difference between an individual’s predicted biological brain age and their chronological age. A positive BAG indicates an “older-looking” brain than expected, serving as a cumulative index of various insults, genetic predispositions, and lifestyle factors that contribute to neurobiological decline. Crucially, in the context of AD, an elevated BAG is hypothesized to reflect more than generalized aging. This metric reflects disease-related neurodegeneration (N) characterized by heterogeneous effects across brain regions, with certain regions exhibiting significantly greater abnormalities relative to age-matched controls. By integrating multivariate features of structural and functional alterations across the whole brain, the metric extends beyond conventional volumetric indices and provides a holistic characterization of pathological burden.

This review explores the BAG as a dynamic, integrated biomarker in AD research. We first present the BAG conceptual framework, highlighting its role in reflecting pathological aging associated with AD, particularly in regions such as the medial temporal lobe (MTL), which is well-established as being prominently affected in AD. The paper details methodologies, contrasting structural MRI models with advanced multimodal fusion strategies. We analyze BAG’s dynamic link to the A/T/N framework and summarize advancements in deep learning for improved prediction and interpretability. Finally, we highlight BAG’s critical clinical utility as a complementary prognostic indicator and a sensitive, adjustable metric for assessing personalized intervention efficacy in precision AD management.

## 2. Conceptual Foundations of Brain Age: Distinguishing Normal Aging from Pathological Aging in AD

**Figure 1:**
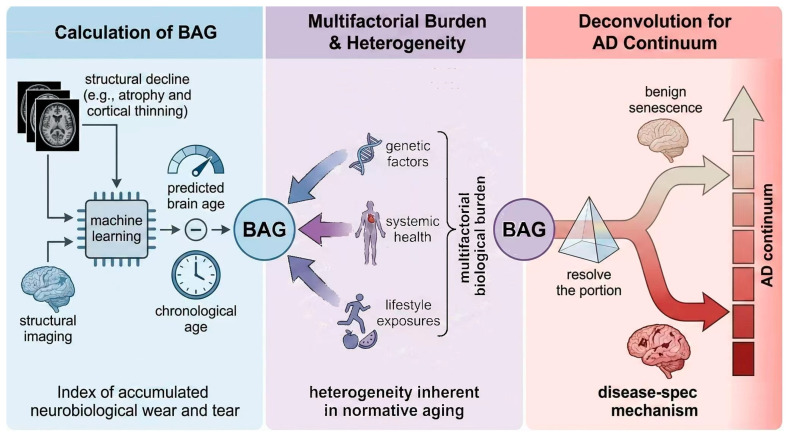
The BAG framework: Calculation, multifactorial burden, and deconvolution within the AD continuum.

The BAG framework represents a data-driven, system-level approach for quantifying individual deviations from expected neurobiological trajectories. It is calculated as the difference between machine learning-derived predicted brain age and chronological age [5]. This scalar measure provides an index of accumulated neurobiological wear and tear, yet its interpretation is fundamentally complicated by the vast heterogeneity inherent in normative aging [6,7]. Large-scale imaging cohorts report considerable inter-individual variability in structural decline, such as atrophy and cortical thinning, even among cognitively unimpaired older adults. This variability suggests that an elevated BAG may reflect a complex integration of multifactorial biological burden rather than simply chronological age [8]. This biological burden is shaped by genetic factors, systemic health, and lifestyle exposures [9,10]. Consequently, models must resolve the portion of the BAG attributable to benign senescence versus that driven by disease-specific mechanisms, providing an important basis for its potential utility within the AD continuum [11]. Figure 1 visually represents the core elements of the BAG framework.

Conceptually, the core challenge hinges on resolving the dual interpretation of BAG: whether it represents a trait-like accumulated risk or a marker of state-like active pathology. From a risk accumulation perspective, accumulating evidence indicates the former, showing that an increased BAG in cognitively unimpaired to early impaired individuals prospectively predicts cognitive decline [12], operating independently of A status—a categorical classification of beta-A plaque burden typically quantified via PET imaging standard uptake value ratios (SUVR), where ‘A-positivity’ (A+) denotes a load exceeding established pathological thresholds [13,14]. This pattern suggests that BAG captures an accumulated neurobiological susceptibility rather than transient disease activity alone.

At the genetic level, this trait-like vulnerability is further supported by inherited risk factors, most notably the APOE4 allele. As the most potent genetic risk factor for sporadic AD, APOE4 impairs A clearance and exacerbates tau-mediated neurotoxicity, neurovascular dysfunction, and neuroinflammatory responses. Collectively, these interacting pathological processes manifest as an elevated BAG decades before clinical onset—particularly during midlife—indicating a genetically driven acceleration of the brain aging phenotype [15,16,17]. Consistent with this interpretation, large-scale international cohorts provide converging evidence for a heritable component of BAG; for example, a genome-wide association study of 28,104 individuals reported a heritability of approximately 27% and identified multiple associated loci, including a prominent signal mapping to the MAPT gene, which encodes the T protein, thereby reinforcing BAG as a biologically grounded marker of AD-related N [18].

When examined longitudinally across the clinical continuum, the influence of APOE4 on BAG is observable well before overt symptom onset. Data from the Alzheimer’s Disease Neuroimaging Initiative (ADNI) cohort (N = 390) with follow-up periods of up to 36 months—and an average of 3 to 4 scans per participant—demonstrate the robustness of this trend. Specifically, longitudinal analyses show that APOE4 carriers—particularly within MCI and AD groups—exhibit significantly higher baseline BAG values and accelerated annual rates of brain aging that outpace chronological progression. These detailed parameters underscore the reliability of our observation that the APOE4 genotype drives an accelerated, rather than static, trajectory of brain aging [19]. Importantly, APOE4 is not deterministically linked to AD diagnosis, and BAG retains strong predictive accuracy for conversion from MCI to AD even when APOE genotype information is unavailable. This dissociation underscores BAG’s role as an integrative downstream phenotype that reflects the cumulative impact of genetic risk rather than serving as a direct surrogate for genotype alone. Notably, inter-individual variability in this relationship may be further shaped by neurovascular integrity and other biological modifiers.

Extending this temporal framework to even earlier preclinical stages, the utility of BAG has also been demonstrated in Subjective Cognitive Decline (SCD), also referred to as Subjective Cognitive Impairment (SCI). Emerging evidence suggests that amnestic SCD represents a critical preclinical phase that may even precede amnestic MCI in the neurodegenerative sequence [20]. A recent study demonstrated that individuals with SCD already exhibit an advanced BAG compared to healthy controls, even when objective clinical assessments remain within normal ranges [14]. This advanced brain age was found to be a potent predictor of future cognitive decline over a 1–2 year period. While traditional neuropsychological scores in SCD populations often remain stagnant due to ‘ceiling effects,’ BAG captures latent neurobiological deterioration that precedes measurable functional loss. Furthermore, this advanced BAG reflects the early impact of A pathology on brain structure, effectively bridging the gap between molecular ‘A+’ status and overt clinical impairment. Together, these findings highlight BAG as an early warning signal capable of capturing subtle neurodegenerative changes during the preclinical “neurodegenerative gap.”

In parallel with this trait-like foundation, the BAG appears to be further sensitive to active disease processes as individuals transition into more advanced stages: longitudinal studies suggest that the brain aging trajectory is accelerated in individuals progressing from MCI to AD dementia [21,22]. At the mechanistic level, accelerated BAG indicates early-stage cognitive decline, potentially reflecting the impact of microglial state transitions on AD-related T pathology [23]. In this context, microglia—the brain’s resident immune cells—shift from homeostatic surveillance to disease-associated phenotypes (DAM). This activation, marked by morphological thickening and cytokine release, creates a neurodegenerative environment that exacerbates structural brain aging, thus providing a biological basis for the predictive value of BAG in clinical settings. Taken together, these findings support the interpretation of BAG as a composite signal integrating lifelong susceptibility with active neurodegenerative processes.

To refine this interpretation at the spatial level, the biological meaning of BAG must move beyond a global scalar index toward distinguishing non-specific senescence from AD-selective vulnerability. Certain brain regions in AD, particularly the MTL (more pronounced as the disease progresses toward MCI and AD) and posterior cingulate cortex (typically emphasized in early preclinical stages), show statistically significant greater atrophy compared with age-matched controls [24]. In this regard, recent advances in normative modeling provide a principled computational framework for spatially resolved brain age estimation [25]. By calculating deviation scores relative to population-derived lifespan trajectories, these models enable the identification of brain regions exhibiting significantly elevated BAG values relative to normative aging patterns, particularly within regions commonly implicated in AD [26]. Crucially, this approach reinforces the concept of pathology-specific brain age, enabling the computational disentanglement of AD-related N from general aging [27]. Crucially, this spatial refinement allows BAG to be meaningfully positioned within the A/T/N classification scheme—a widely accepted biological system that categorizes AD biomarkers based on the presence and progression of A, T, and N. In this context, BAG serves as an informative indicator of the component, tracking disease progression more closely than chronological age.

## 3. Structural MRI and Brain Age in AD

Originating from machine learning models trained on sMRI data from healthy cohorts, BAG serves as an integrated metric of brain health and accelerated biological aging [28]. Early research established the fundamental relationship between sMRI features and BAG. The model’s prediction is based on quantifying neuroanatomical integrity, including whole-brain volume, cortical thickness, and hippocampal volume [29,30]. Consequently, an elevated BAG is considered a potential indicator of pathological vulnerability and tends to be higher across the AD spectrum, from MCI to overt dementia [12,31,32].

While total brain volume and global gray matter volume are useful for generating the initial estimate of BAG, they primarily reflect general age-related brain shrinkage rather than specific AD pathology. As such, these global metrics offer limited specificity in distinguishing AD from typical aging [33]. In contrast, BAG’s predictive power in AD is mainly driven by local neuroanatomical features. These features align with the disease’s characteristic patterns of degeneration. Specifically, atrophy in the hippocampus and thinning of the cortical mantle—particularly in later-stage affected regions such as the temporal and parietal lobes,—are key contributors to the accelerated BAG observed in AD cohorts [34,35]. These localized changes offer more specific indicators of AD pathology than global volume measures, supporting the BAG framework in representing the spatial distribution of N.

Higher BAG values often appear early in the AD continuum, enhancing its clinical utility as a prognostic marker. Longitudinal evidence suggests that accelerated rates of hippocampal atrophy are most pronounced in MCI subjects who subsequently progress to AD, indicating that the acceleration is driven by the pathological process, not just the state of impairment [36]. Moreover, studies investigating the longitudinal increase in BAG have found that BAG acceleration is evident in the MCI group, even potentially preceding substantial hippocampal volume loss [37,38]. This suggests that the subtle, widespread microstructural decay and early atrophy captured by the sMRI-BAG model serve as a sensitive marker. They indicate the initial shift towards a pathological trajectory. The early detection of an accelerated BAG in MCI provides a critical window for intervention, positioning it as a useful biomarker for patient stratification and monitoring efficacy in early-stage AD clinical trials.

## 4. The Value of Multimodal Brain Age in AD

The value of multimodal brain age, which integrates data from multiple neuroimaging techniques (such as MRI, PET, and fMRI), has emerged as a promising approach to better understand the progression and early detection of AD [39,40,41,42]. By combining the strengths of each imaging modality, this approach offers a more comprehensive picture of brain health than single-modality methods.

The temporal divergence between functional and structural decline represents a critical frontier in AD characterization. Recent evidence suggests a phenomenon called “structure-function decoupling.” In this case, functional brain age estimates, derived from fMRI or Electroencephalography, may paradoxically appear preserved or even “younger” during the preclinical A+ stage, a phase in which A-beta accumulation has begun but cognitive performance remains largely intact, and compensatory neural mechanisms may temporarily maintain functional activity. This contrasts with the accelerated aging observed in structural models [43,44]. Millar et al. [45] observed this biphasic response, hypothesizing that early hyperconnectivity or compensatory network reorganization masks the latent pathology before synaptic failure precipitates a rapid functional decline. Consequently, relying solely on sMRI based brain age risks missing this prodromal window, whereas functional biomarkers capture the dynamic, non-linear synaptic alterations that define the disease’s earliest, most malleable phase.

While structural atrophy indicates neuronal death, metabolic dysfunction is the harbinger of synaptic silence. PET-derived brain age models have shown promising sensitivity in detecting “synaptic aging,” sometimes identifying abnormalities years before volumetric loss becomes statistically significant [46]. As highlighted by Lee et al. [12], metabolic brain age serves as a useful proxy for synaptic density, particularly in the posterior cingulate and precuneus—regions metabolically devastated in early AD. This metabolic sensitivity allows clinicians to stratify patients who may have “structurally young” but “metabolically old” brains, identifying a subgroup at imminent risk of conversion to dementia that would otherwise be overlooked by morphological screening alone.

Traditional brain age models often neglect the “cabling” of the brain, yet white matter integrity offers distinct insights into the vascular and disconnection components of AD [47,48]. DTI fills this gap by quantifying microstructural degradation—specifically reductions in fractional anisotropy (FA) and increases in mean diffusivity (MD)—which often reflect comorbid cerebral small vessel disease (cSVD) rather than pure A pathology. Research in Stroke [49] indicates that incorporating DTI metrics refines brain age estimation by disentangling vascular aging from neurodegenerative atrophy. By accounting for white matter hyperintensities and tract-specific degeneration, multimodal models can better attribute “brain aging” to its specific vascular or neurodegenerative drivers, offering a more personalized risk profile.

The true power of multimodal brain age lies not merely in collecting diverse data, but in how that data is integrated. Simple “data concatenation” often fails to capture the complex, non-linear interactions between modalities. Advanced deep learning fusion strategies, such as the feature-level fusion layers or attention-based mechanisms, which appear to outperform basic concatenation [48,50,51]. These architectures allow the model to learn cross-modal representations, dynamically weighting the importance of metabolic versus structural features for each individual. This transition from simple feature concatenation to integrated deep fusion allows for a more comprehensive modeling of the regional and functional heterogeneity observed in AD. It yields a composite “biological brain age” metric that demonstrates improved predictive performance for clinical progression compared with individual features alone. The integrated framework for multimodal brain age, which leverages a range of neuroimaging techniques and advanced fusion strategies, offers a more integrative view of AD pathology and may assist in patient stratification. As summarized in Table 1, different neuroimaging modalities capture distinct biological dimensions of brain aging, providing the biological basis for multimodal fusion and influencing how BAG reflects accumulated vulnerability versus active disease processes across the AD continuum.

While these advanced fusion strategies substantially enhance the biological and predictive validity of multimodal brain age, translating them into practice still introduces several methodological and clinical challenges. First, data harmonization across imaging modalities and acquisition sites remains non-trivial, as modality-specific noise characteristics, scanner effects, and preprocessing pipelines can introduce systematic biases into BAG estimates [52]. Second, multimodal integration substantially increases computational complexity, requiring larger sample sizes, greater model capacity, and careful regularization to avoid overfitting—constraints that may limit scalability in typical clinical cohorts. Finally, clinical applicability remains an open challenge, as not all patients undergo comprehensive multimodal imaging, and the interpretability of complex fusion models may hinder routine clinical adoption. Addressing these challenges is therefore critical for translating multimodal BAG from a research framework into a clinically viable biomarker.

## 5. The Association Between Brain Age and Typical Biomarkers of AD

The brain age represents neuroimaging biomarker for brain health, potentially offering insights neurodegenerative diseases like AD. In this context, it is critical to correlate imaging-based brain age with biological gold standards, such as the A/T/N framework [53], which classifies AD biomarkers based on A, T, and N. This approach not only helps validate the pathophysiological basis of brain age but also enhances its utility in understanding the disease’s progression and early detection.

The relationship between the BAG and AD’s pathology is governed by a distinct nonlinearity within the A/T/N framework, challenging the assumption of a linear progression. Recent evidence indicates that in the early stage of A deposition without significant tauopathy (A+T-), structural brain age may remain stable or even appear paradoxically “younger.” This stage is characterized by A deposition without significant tauopathy, and the effect may result from compensatory hyperconnectivity. It is only with the onset of “T+” (T pathology) that BAG appears to show a marked acceleration, suggesting that while A sets the stage, T dissemination could play an important role of the neurodegenerative “aging” signal. This biphasic trajectory suggests that BAG is not a uniform marker of disease duration but a dynamic readout of the transition from Aosis to T-mediated synaptic failure [54].

BAG appears to be closely associated with the pathogenesis of AD, potentially driven by chronic neuroinflammation. Neuroinflammaging—the age-related chronic inflammatory state, may represent a transitional process between normal senescence and the onset of age-related neurodegenerative disorders like AD [55]. While neuroinflammation is a pervasive feature of AD, its intensity may vary across disease stages; interestingly, some evidence suggests that the association between classical neuroinflammatory markers (e.g., microglial activation) and AD pathology could be more pronounced in relatively younger AD patients compared to the oldest populations [56]. Regardless of this age-dependent variation, the cumulative inflammatory burden accelerates biological brain aging, suggesting that chronic inflammation may contribute to increased vulnerability to, or faster progression of AD [57].

A key methodological limitation in many earlier brain age models is the inadvertent inclusion of A+, cognitively normal elders in the normative training set, which may introduce bias in the estimation of expected aging trajectories. These individuals, while asymptomatic, harbor latent pathology that subtly alters the model’s baseline of “healthy aging,” thereby reducing its sensitivity to early disease deviations. Recent studies suggest that models trained exclusively on rigorously screened “A-” controls (A-negative) may generate a “Corrected Brain Age” that is more sensitive to the earliest deviations in AD. This refinement may help transition brain age from a general research concept toward a clinically useful tool for detecting the subtle “neurodegenerative gap” in the preclinical phase [58].

Taken together, the practical utility of the BAG must be contextualized within the recently revised 2024 National Institute on Aging and the Alzheimer’s Association (NIA-AA) criteria [59], which transition from the descriptive A/T/N system to a biologically grounded A, Phosphorylated T 1, T pathology 2, N, Inflammation, Vascular injury, and Susceptibility/Genetic risk (AT1T2NIVS) hierarchy. This diagnostic shift is significantly empowered by high-performance blood-based biomarkers; notably, plasma p-T217 has demonstrated accuracy comparable to CSF and PET imaging in differentiating Aβ+ from Aβ- individuals [60]. This advancement facilitates a transformative synergy: while p-T217 provides a high-specificity confirmation of the underlying molecular pathology (‘Core 1’ status), BAG serves as a critical readout of the resulting neurodegenerative ‘toll’ on brain structure and function (‘Core 2’).

The interpretation of this ‘toll’ further diverges between the neurobiological criteria (e.g., NIA-AA) and the clinical-neurobiological criteria (e.g., IWG) [61]. Under the neurobiological framework, which defines AD independent of symptoms, an elevated BAG acts as an objective ‘Core 2’ marker contributing to diagnosis even in asymptomatic stages. In contrast, the IWG criteria mandate concurrent clinical evidence for a formal diagnosis. Within this framework, BAG is repositioned as a risk-stratification or staging marker; a high BAG in a cognitively normal individual identifies a ‘high-risk’ preclinical state rather than AD itself.

Consequently, integrating these frameworks enables a multidomain profiling approach. For instance, ‘Core 1’ positivity (p-T217+) alongside a stable BAG may reflect high brain resilience or an incipient phase. Conversely, a rapidly accelerating BAG in p-T217+ patients pinpoints a window of aggressive N. By incorporating functional modalities, BAG transcends mere atrophy measurement; it captures metabolic and connectivity disruptions that precede structural loss, reflecting the early toxic effects of A and T. Mechanistically, BAG serves as a dynamic indicator of how molecular pathology compromises brain resilience and accelerates the aging trajectory. To bridge the gap between biological aging and clinical practice, we have provided Table 2, which contrasts the interpretation of BAG within the frameworks of NIA-AA and the IWG criteria.

## 6. An Overview of Deep Learning-Based Brain Age Models for AD Prediction

The pursuit of an accurate and generalizable ‘brain age’ biomarker has contributed to notable progress in neuroimaging, with deep learning playing an increasingly important role. Initially, Convolutional Neural Networks (CNNs) were central to these efforts, excelling in 3D sMRI analysis [62,63]. Early CNN models were inspired by established image classification architectures like VGG-16. They could map complex structural changes, such as gray matter density and white matter integrity, directly to chronological age. These models showed a notable reduction in Mean Absolute Errors (MAE) [12]. CNNs excelled in automatically extracting hierarchical, translation-invariant features from raw volumetric data, with minimal preprocessing required, allowing them to capture intricate patterns of brain morphology [64]. This capability has been particularly useful in brain age prediction tasks, where understanding subtle structural variations is crucial. For instance, He et al. [65] leverages a multi-stream architecture to integrate diverse MRI features across the lifespan. By incorporating attention mechanisms, it effectively captures spatial and temporal patterns, enhancing brain age prediction accuracy and interpretability. Similarly, Jiang et al. [66] utilizes structural MRI parcellation to segment the brain into regions of interest. It then applies convolutional layers to extract spatial features from these regions, enabling accurate brain age predictions by learning patterns across different brain areas.

Despite their strengths, CNNs face limitations in modeling long-range dependencies and global connectivity patterns, which are essential for understanding AD. In AD, subtle and widespread disruptions across distant brain regions play a crucial role in early diagnosis. However, CNNs, with their localized receptive fields, struggle to effectively capture these complex, long-range interactions. To address this limitation, recent advancements have introduced more sophisticated models, such as transformers (including Vision Transformers (ViTs)) and Graph Neural Networks (GNNs). Unlike CNNs, ViTs reformulate 3D brain MRI volumes into a sequence of flattened ‘patches’ or visual tokens. By employing multi-head self-attention mechanisms, ViTs can compute the relative importance and spatial dependencies between all anatomical patches simultaneously, regardless of their physical distance. This structural shift allows ViTs to transcend local processing, excelling at capturing the global relationships and long-range dependencies necessary for modeling the inter-regional connectivity disruptions and systemic network failures observed in AD [39,67,68]. A notable example of this model evolution is dual-stream fully convolutional residual networks (ds-FCRN), which combines CNNs’ local feature extraction with transformers’ ability to capture long-range dependencies, enhancing predictive accuracy and providing a more comprehensive understanding of early-stage AD [69].

**Figure 2:**
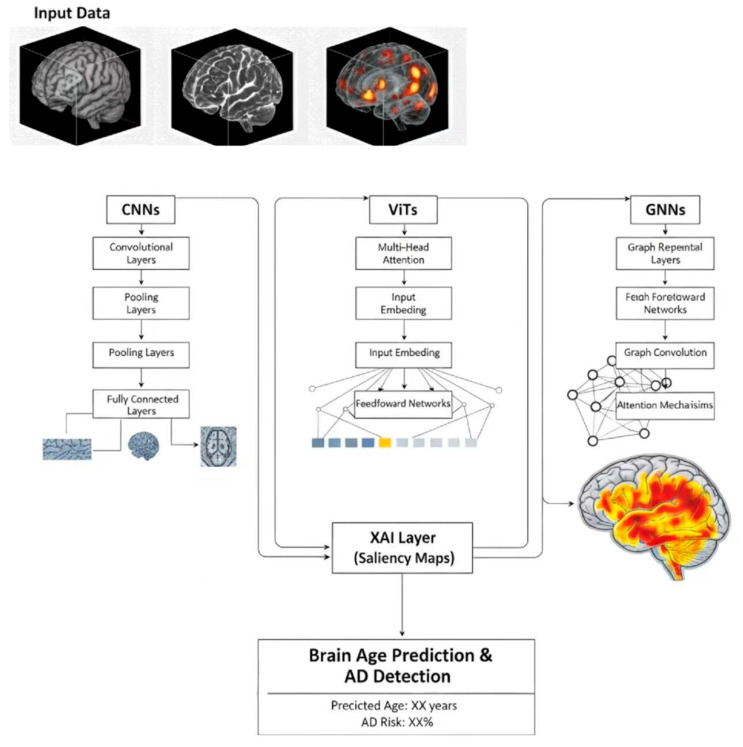
The architecture of the brain age prediction model for AD detection.

Similarly, GNNs have gained traction in the study of brain networks, as they are particularly well-suited to data structured as graphs [70]. In this framework, each brain region is represented as a node, and the edges represent the connectivity between them. By leveraging GNNs, researchers can more accurately capture the brain’s network-level disruptions, which are crucial for understanding the early stages of AD. These models improve the predictive accuracy of brain age. They also provide a more nuanced understanding of how different brain regions interact. This offers deeper insights into the underlying pathophysiology of neurodegenerative diseases such as AD. An important example of GNN application is the Interpretable Attention based Graph Convolutional Network (IA-GCN) model [71], which integrates attention mechanisms into GNNs to enhance interpretability and improve disease prediction. Together, these innovations represent progress in the development of brain age models, enhancing their ability to capture both structural and functional disruptions. Such capabilities are essential for characterizing the neurobiological impact of AD pathology and providing a more nuanced assessment of disease progression. Figure 2 illustrates the architecture of the brain age prediction model.

Despite the success of deep learning in AD prediction, one major hurdle remains: the “black-box” nature of these models. Clinicians, who are ultimately responsible for making treatment decisions based on these models, need to trust the predictions provided by AI. This issue is compounded by the complexity of the brain, where multiple regions interact in ways that are difficult to interpret. To address this, Explainable AI (XAI) techniques [72,73], particularly saliency maps, have been developed to provide insights into the model’s decision-making process [74]. From a technical perspective, saliency maps are generated using gradient-based attribution methods, which compute the partial derivatives of the model’s output (predicted brain age) with respect to each input MRI voxel. By backpropagating this importance signal through the network, the method assigns a specific weight to each voxel that reflects its relative influence on the final prediction. These weights are then aggregated to identify ‘salient brain regions’—key anatomical areas, such as the hippocampus, where the model detects the most significant features during its evaluation. This approach not only provides transparency but also allows clinicians to visually assess whether the model is focusing on areas of the brain that are known to be affected by AD. By improving the interpretability of the model, XAI can bridge the gap between AI research and clinical application, helping to address trust concerns faced by healthcare providers.

## 7. The Predictive Value of Brain Age for Cognitive Decline and Clinical Progression

A key distinction in brain age research lies between static BAG and dynamic measures of aging acceleration, such as Pace of Aging (PoA). Static BAG provides a snapshot of how much older an individual’s brain appears relative to their chronological age. While informative, it has limitations when it comes to forecasting future decline. Dynamic measures, captured by the longitudinal change in BAG—also referred to as the PoA, particularly focusing on the rate of change over time, provides a more dynamic perspective. This dynamic perspective is critical for capturing ongoing. Longitudinal analyses involving 183 participants and a total of 677 MRI scans (median of 4 scans per participant) indicate that MCI patients tend to exhibit an accelerated PoA compared to healthy controls over an average follow-up period of 2.0 years, suggesting that the stage of MCI is when active, pathological brain aging is most pronounced [38]. Furthermore, the clinical relevance of PoA is underscored by its correlation with neuropsychological decline and shifting plasma biomarkers [22]. Specifically, PoA mirrors the dynamics of neurofilament light chain—a structural axonal protein indicating general N, and p-T (which specifically tracks AD-related T pathology). BAG shows a significant increase over time in both amnestic and non-amnestic sporadic Early Onset AD. This supports its potential value as a robust marker for tracking disease severity and evolution [75]. Therefore, PoA acts as a sensitive, quantitative measure of the rate of biological deterioration, offering enhanced prognostic sensitivity over static BAG measurements.

Specifically, the BAG is considered a promising biomarker that may reflect pathological aging rather than healthy aging. Studies have reported that a larger BAG is associated with lower cognitive scores across various assessments, including MMSE and MoCA [76,77]. BAG shows potential for identifying early cognitive impairment, particularly in conditions where cognitive decline is subtle [77]. This highlights BAG’s potential utility in providing an objective, quantitative measure of brain health. BAG may complement, and in some cases precede, clinical scales in detecting deficits. Clinical scales are often limited by ceiling effects and may miss subtle, non-symptomatic structural vulnerability [76].

Both the BAG and Cognitive Reserve (CR) [78] are fundamentally conceptualized as residual measures that quantify disparities between expected and observed outcomes in aging [8,79]. The BAG is the residual error of a ML model predicting chronological age from neuroimaging features, serving as an integrated biomarker of structural aging pathology. Conversely, CR is often operationalized as the cognitive residual—the variance in cognitive performance unexplained by measured brain pathology [80]. A critical area of research is determining whether the BAG can explain the variance traditionally attributed to CR. When BAG is used as the primary measure of neurobiological vulnerability, the BAG-derived residual indicates the cognitive performance not explained by biological brain age. This residual becomes the operational index of CR [79]. This approach allows researchers to isolate and quantify an individual’s cognitive resilience against systemic biological aging, moving beyond simple proxies like education or lifestyle. However, some studies suggest that while BAG captures the brain maintenance aspect of aging, the CR residual may encompass factors (like network efficiency or compensation) that are not fully reflected in BAG, indicating that BAG acts as a refinement rather than a complete replacement for the existing CR concept [81,82].

## 8. Brain Age and Its Significance in AD Interventions

An important question surrounding brain age is whether it represents irreversible damage accumulation or if it can be dynamically adjusted through lifestyle interventions. Recent longitudinal evidence suggests the latter, positioning that the BAG may serve as a potentially modifiable biomarker of biological aging. Comprehensive lifestyle interventions, particularly those incorporating regular physical activity and improved diet, shown promising results in slowing the rate of BAG progression in older adults or individuals at elevated risk for cognitive decline [10]. For instance, studies have found that the reduction in BAG following structured exercise regimens may align with improved clinical outcomes, demonstrating that decelerating brain aging is a tangible goal linked to functional recovery [83]. The underlying mechanism may involve systemic health changes: reductions in adverse body composition markers, such as visceral adipose tissue (VAT), are associated with a more favorable BAG trajectory, highlighting a putative link between metabolic health and brain aging rate [84]. Furthermore, robust dietary interventions, such as adherence to a high-polyphenol Mediterranean diet combined with physical activity, have shown potential to mitigate age-related brain atrophy—a primary structural contributor to an increasing BAG—by protecting structural integrity [85]. These findings reposition BAG from a static predictor of vulnerability to a dynamic metric for monitoring brain health trajectories and intervention efficacy.

The biological basis for such targeted interventions is further illuminated by large-scale proteomic evidence. A study of 43,616 participants from the UK Biobank by Wang et al. [86] developed organ-specific proteomic clocks, revealing that individuals categorized as ‘brain-agers’—those with a brain age 1 standard deviation above the mean—face a 2.5-fold increased risk of progressing to AD. Crucially, the discovery that nearly 20% of the population exhibits accelerated aging predominantly in a single organ, such as the brain, underscores the necessity of brain-specific monitoring. These findings suggest that an elevated BAG can isolate brain-specific senescence from systemic aging, providing a robust molecular rationale for using BAG as a precision monitoring tool.

The utility of the BAG could be important for personalized medicine in because it provides a quantitative window to better understand treatment heterogeneity-the phenomenon where patients with the same clinical diagnosis respond differently to interventions. By tracking the dynamic reduction or stabilization of an individual’s BAG during a therapeutic trial, clinicians may obtain an objective, personalized supplementary metric to assess a patient’s neurobiological aging rate. This capability supports a more nuanced approach to disease management, allowing clinicians to refine lifestyle protocols or exercise intensity based on individualized biological feedback. While further large-scale validation is required to establish clinical thresholds, this shift represents a critical step toward moving AD management toward precision medicine.

## 9. Conclusions, Current Limitations, and Future Trajectories

In conclusion, the BAG metric emerges as a promising systems-level complement to traditional, single-marker assessment to an integrated, dynamic biomarker in AD. The evolution from a static BAG snapshot to the longitudinal PoA offers enhanced insights into disease staging and can better reflect the progression of AD pathology. This value is supported by studies linking BAG to the A/T/N framework, particularly T and neuroinflammation. However, several critical barriers must be addressed. Methodological limitations, including poor model generalizability across diverse populations and the high cost of essential multimodal data, constrain widespread clinical adoption. Furthermore, the “black box” nature of deep learning models highlights the need for further development of XAI to enhance clinical trust. Addressing these challenges requires a transition from retrospective observations to prospective validation. Large-scale initiatives such as the UK Biobank and ADNI have demonstrated BAG’s predictive potential, and ongoing efforts like ADNI-4 aim to establish standardized, normative aging templates across diverse populations—a crucial step toward enabling individual-level diagnosis. Looking forward, future research should prioritize three key areas to maximize BAG’s clinical utility: (1) Standardizing calculation methods and exploring the feasibility of normative clinical cutoffs for BAG and PoA; (2) Integrating multi-omics data (genomics, proteomics) to precisely decompose the variance within the BAG; and (3) Utilizing BAG as a supplementary outcome in prospective, multi-center studies to further evaluate its utility in monitoring interventions and support the progress towards personalized medicine in AD.

## Figures and Tables

**Table 1:** Multimodal comparison of BAG estimation.

Modality	Biological Dimensions	BAG Temporal Profile	Key Strengths for BAG Modeling	Major Limitations	Clinical Value
sMRI	Macrostructural atrophy and gray matter loss.	Late/Persistent: Reflects cumulative, irreversible damage.	High reproducibility; strong age signal; robust generalization; interpretable anatomy	Limited sensitivity to early synaptic or molecular changes	Quantifies trait-like BAG reflecting cumulative neurodegeneration and long-term cognitive risk.
fMRI	Functional network dysregulation and BOLD dynamics.	Early/Preclinical: Detects functional aging before atrophy.	Detects microstructural damage preceding cortical atrophy; sensitive to aging and vascular injury	Complex modeling (e.g., crossing fibers); susceptible to eddy currents.	Detects early microstructural aging, enhancing BAG sensitivity before cortical atrophy.
DTI	White matter microstructural integrity (axonal damage, myelination; early N)	Intermediate: Captures the breakdown of structural connectivity.	Captures early network disruption and compensatory mechanisms	High inter-site variability; lower longitudinal stability	Captures state-dependent functional aging and network vulnerability within BAG.
PET	Molecular pathology and metabolic dysfunction (A/T) and Glucose metabolism	Very Early to Late: A (early) T/Fluorodeoxyglucose (progressive).	Pathology-specific; direct mechanistic anchoring	High cost; radiation; limited scalability	Anchors BAG to specific molecular pathology, improving etiological interpretation.
EEG	Synaptic oscillations and cortical excitability.	Dynamic/Early: Reflects real-time neurophysiological state.	High temporal resolution; cost-effective; portable.	Poor spatial resolution; high sensitivity to ocular artifacts.	Reflects synaptic and oscillatory aging, enabling dynamic BAG monitoring.

**Table 2:** Comparison of BAG Interpretation within NIA-AA and IWG Diagnostic Frameworks.

Feature	NIA-AA Research Framework	IWG Criteria
Core Definition of AD	Biological construct: AD is defined by the presence of A and T, regardless of clinical symptoms.	Clinical-biological construct: AD requires the presence of both A and T, and objective clinical impairment.
Role of BAG	Serves as a continuous proxy for N to stage disease severity.	Functions as a prognostic biomarker to assess the risk of progression in asymptomatic individuals.
Cognitively Normal Status	high BAG reflects early pathological neurobiological toll.	high BAG indicates an increased probability of future clinical conversion
Clinical Utility	Used for tracking individuals’ position along the biological continuum.	Emphasizes BAG as a marker of vulnerability that informs clinical prognosis rather than defining the disease itself.

## Data Availability

No new data were created or analyzed in this study.
